# Notes and Comments

**Published:** 1924-12

**Authors:** 


### NOTES AND COMMENTS.

### "THE HOSPITAL AND HEALTH REVIEW."

WITH this issue THE HOSPITAL AND HEALTH REVIEW ceases publication. A brief note on the history of the paper seems not out of place. THE HOSPITAL was founded in 1886 by the late Sir Henry Burdett, who edited it until his death in 1920. To an unusal degree the paper was identified with its editor, and the editor with his paper; and though there were many who did not agree with Sir Henry Burdett's views, they were of a nature to compel attention. It was inevitable that after his death THE HOSPITAL, in its weekly form, should be discontinued. Its place was taken by THE HOSPITAL AND HEALTH REVIEW, which now makes its last appearance. It may be that the attempt we have made to combine, in one paper, these two divergent aspects of public medicine was too ambitious for the times. If that is so, we do not regret our endeavour, and are satisfied to hope that time may remove the impediment to this "marriage of true minds."

---

### A HAPPY RETURN.

MR. NEVILLE CHAMBERLAIN'S return to the Ministry of Health is a peculiarly happy result of the General Election. Without in any way disparaging his predecessors, it is the merest truth to say that he is the only one of far too long a line who has made any impression upon the obstinate problem of housing. The scheme with which Dr. Addison's name is associated was a terribly costly failure; Mr. Wheatley's scheme, if ever it were to get really into operation, would fail as disastrously and at least as expensively. Present indications, however, are that local authorities will fight shy of the Wheatley Act—they prefer the lower bonus and smaller risks of the Chamberlain Act. That measure has already given us a large number of houses, and in the course of another few months will give us thousands more; it is true that the Wheatley Act has not had time to give us any, but there is not even a sign that it will give us any to speak of. We have yet to see what steel and concrete can do in the way of producing houses quickly—there is not much reason to hope that either method will be much cheaper than the other. We are entitled to brush aside the argument that houses built to sell do not help people who are compelled to pay rent, since it is obvious that every new house helps to ease the scarcity. Meanwhile it is highly encouraging to learn from Mr. Chamberlain that his Act has already provided nearly 74,000 houses of an annual value below £26, mainly built by private enterprise—the only truly economic method of solving the difficulty.

---

### THE PUBLIC MEDICAL SERVICES.

MR. CHAMBERLAIN has lost no time in announcing that his work upon the housing problem is not to exclude from his purview the other big health questions which the Ministry was brought into being to tackle. He fairly says that he has entered into office in more favourable conditions than his more recent predecessors, and that it should now be possible with some confidence to plan and scheme over an extended period, and that it is only by so doing that steady and consistent progress can be made. Pointing to the need for large central institutions to provide adequate facilities for a cluster of towns, around Birmingham for example, Mr. Chamberlain points out that if we are to deal with the problems of making the maimed whole in a statesmanlike way we have to forget what are the boundaries of our local government areas. It is his hope before he leaves office to introduce some new plan of co-ordination and specialization in these great medical services. It would almost seem as if the report of the Consultative Council on the development of medical and allied services is coming out of its pigeon-hole. It is certain that the Commission on National Health Insurance will have some interesting recommendations to make on the same question.

---

### REDUCING TEMPTATION.

WHILE it is true that people can be made neither moral nor sober by Act of Parliament, experience shows that wise legislation helps substantially to keep them out of mischief. There are fewer public-houses, they are open for a shorter time than formerly, and there has been a gratifying diminution in drunkenness. Drug addiction is one of the worst perils of modern life, and we are reducing it by going to the root of the trouble by making it difficult to obtain drugs which injure both body and mind. In 1920 we passed the Dangerous Drugs Act in fulfilment of the International Opium Convention of 1920, and the Home Office has just issued some very encouraging figures of the results as regards morphine and heroin. In the year the Act was passed we manufactured 644,000 ounces of morphine and its salts; last year the production sank to a trifle above 88,000. In the same year we produced 76,000 ounces of heroin and its salts; last year the production was well under 12,000. These heavy reductions are very significant, and we seem now to be well on the way to a production which shall suffice for legitimate medical needs and no more. The power to do ill deeds makes ill deeds done, and the more difficult it is made to obtain these deadly drugs the sooner will their right use supersede the abuse which has caused so much crime and misery.

---

### HEALTH IN THE SCHOOL.

SIR GEORGE NEWMAN'S Annual Report of the Health of the School Child (Stationery Office, 2s. net) records the medical inspection of 1,754,919 elementary school children during 1923, of whom 19.4 per cent. were found to be suffering from some more or less serious physical defects. Two-thirds of the whole school population are suffering from dental disease, and during last year the enormous number of 135,269 children were ordered spectacles by the school oculists, of whom some 15,000 failed to obtain them. Mr. Corkery, the School Medical Inspector, has some significant things to say of the decadence of the rural school child, whose general physique, appearance and stature show a steady and manifest decline. Breeding from the unfit, bad training, intermarriage and the large size of families are some of the chief factors contributing, in his opinion, to the decline, together with the lack of sufficient and suitable foods. Country children have often to walk two or three miles to their school; they take with them a very inadequate lunch, and their return journey is usually followed by tea and bread and margarine, with perhaps fried potatoes or a vegetable stew. This is a bad state of affairs which is already being remedied at certain schools, where the preparation and service of a nourishing midday meal is practically a part of the educational routine. On the whole the Report is hopeful of future possibilities. "It is not," writes Sir George Newman, "that there are great extensions or exceptional achievements to record for 1923, but, owing to somewhat less financial stringency, and owing to consolidation and a fuller appreciation of the value of the service in regard both to the treatment and the prevention of disease among children, there is a promise of wide and fruitful development."

---

### THREE MILLION LEPERS.

IT is disquieting to learn from a speech by Sir Leonard Rogers at the Institute of Public Health that with the opening up of communications and trade routes the terrible scourge of leprosy is extending in tropical Africa. He points out that the whole history of its spread is that of an insidious and slowly communicable disease carried by armies and the immigration of infected races to previously immune countries. The prevalance of the disease is connected with damp heat, and the hot areas of the tropics are most seriously affected. The Annual Report of the Mission to Lepers, a Society which has for fifty years carried on a quietly heroic work, is also convinced of the need of organised effort in combating the disease in Africa, where an American missionary estimates that there are 20,000 lepers in his district alone. While acknowledging the remarkable results achieved by the new treatment of leprosy, the Mission naturally regards it as premature to speak of a certain cure for the disease, but measurable success has been secured in at all events retarding it. The readiness of sufferers in early stages to come forward is a particularly encouraging feature. Many inmates of the Mission's Homes have been discharged as being apparently free from active or other symptoms of leprosy, though there is a certain reluctance on the part of patients to leave institutions where so much has been done for them. Destitute and wandering lepers are a dangerous source of infection, and their segregation is a matter of difficulty. Of the 3,000,000 lepers in existence, one-tenth are citizens of the British Empire, and it is not surprising that the appeal of the Mission to Lepers everywhere falls on sympathetic ears.

### REFORM OF THE POOR LAW.

LORD GEORGE HAMILTON has written a valuable letter to the *Times* in which he reminds the country of the recommendations of the Poor Law Commission, of which he was the distinguished chairman, amd of the promises of reform, following on those recommendations which have so long remained dormant. Now, says Lord George, is the moment for such an effort as is necessary; and although, he continues, the opposition to change will at the outset be noisy, it will soon die down when the indisputable benefits of the changes manifest themselves. It may prove to be so. The noisy opposition may certainly be reckoned upon, especially if the boundaries, which Mr. Neville Chamberlain says that we must forget are going to be altered, as indeed they must be if reform is to be on the grand scale.

---

### A MUSEUM OF TROPICAL MEDICINE.

APART from the possibility of next year renewing our acquaintance with the Tropical exhibit at Wembley referred to above, it is pleasant to learn that the Museum of the new London School of Hygiene and Tropical Medicine will contain a spacious section devoted to this interesting side of health study. Such a museum should prove of great value not only to British, Colonial and foreign students, but to the general public. It is well-known that the Director of the School, Dr. Andrew Balfour, has long been an enthusiast for this graphic form of teaching. He was Chairman of the Sub-Committee of the Tropical Health Section at Wembley, and it may be anticipated that the museum of the School will be developed on the same attractive lines, and will prove a constant source of interest and study to visitors from all parts of the kingdom and from overseas.

---

### "RATS!"

WHEN "winter comes" one of the few satisfactions of the season is that there are fewer rats in this island. The annual "Rat Week" has been included in the past month, and we trust that the bags have been considerable. Unhappily, a census of rats is impossible, and we have no definite means of estimating the extent of the problem of extermination. The killing of a hundred thousand or so probably makes no substantial difference to the many army corps of brown rats that are encamped all over the country, taking toll of the farmer's grain and other crops, spreading disease and generally making themselves a nuisance. They have now taken to destroying nesting birds on Ailsa Craig, where, during the past season, they have committed serious ravages. It might have been supposed that the comparative inaccessibility of Ailsa Craig would have saved it from invasion, but rats have been known to be present there for a generation past. We cannot allow the solan goose to be exterminated by unworthy vermin, but it is no longer to be found in England—it deserted Lundy Island some time ago. The Society for the Protection of Birds is about to start a campaign which it is hoped may rid the famous rock at the mouth of the Clyde of its unwelcome visitors, or, at the least, sensibly diminish their numbers and their possibilities of harm. The isolation of the spot and the difficulty of recruiting to fill the depleted ranks of the brown rascals ought to make the possibilities of success very promising.

### DANGERS OF DUST.

WHEN we recall the number of diseases and minor troubles, such as sore throat, traceable to the inhalation of dust in an ordinary or a special form, we need feel little surprise that a year which has been wet enough to "lay the dust" much more effectually than usual, should have been the healthiest year ever known. The general death-rate of the September quarter was the lowest on record; so was the infant death-rate. On the other hand, the birth-rate was the lowest recorded in any third quarter except during the war. It would thus appear that it is better to have little dust than little sun—darkness is a less deadly enemy than dust. Abundant sunshine would have given us a far greater amount of dust. As the science of road-making improves there ought to be a notable decrease in the volume of dust; we could then afford hot summers without fear of swallowing millions of dust-microbes, especially if we can abolish the uncovered dust-cart which scatters mischief all over us every time there is a good puff of wind. The long season of wet and damp has produced an unusually large crop of rheumatism—another of our characteristic national diseases, and not the least potent in loss of physical efficiency and working power. Thus in dry weather we swallow dust; in wet weather we are racked by rheumatism. We can hardly expect to eliminate either of these troubles, but it is certain that they can be much reduced since, although we cannot command the elements, we can do much to counteract their efforts to make our earthly pilgrimage brief and troubled.

### FOOD VALUE OF THE BANANA.

AT the present time the food value of a given substance is largely determined by its vitamin content, and if it can be shown to contain all the vitamins already identified, it possesses the wherewithal to cover a multitude of sins. The banana, by the way, has no sins to cover unless it be that its cost is still capable of reduction. It contains from 19 to 20 per cent. of sugar and starch, from 4 to 5 per cent. of protein, and from 0.5 to 1 per cent. of fat. Its pleasant taste is another advantage on which it is superfluous to expatiate, and we now learn from investigations recently carried out by Dr. Eva Sopp, at the Kapp Laboratory in Norway, that it contains not only B-vitamins and C-vitamins, but also the A-vitamin, the absence of which in the food of the growing mammal causes a very serious disease of the eye, known as xerophthalmia. She fed about 25 young rats on a diet deprived of A-vitamins, and after they had lost weight and developed xerophthalmia, she divided them into three groups, each of which was given a different quantity of banana. But whether the rats were given only 1 gramme of banana daily or as much as they could eat, the results were practically the same—after about a fortnight they began to gain weight steadily, and the eyes resumed their normal appearance. Applying the results of these investigations to the human being, Dr. Sopp argues that if 1 gramme of banana is sufficient to provide a rat, weighing 50 grammes, with an adequate supply of A-vitamins, a three-year old child would receive the equivalent amount of A-vitamins if given four or five bananas a day. The child to whom cod liver oil is anathema surely owes Dr. Sopp much for proving that the banana is not merely tasty, but is the repository of a rich store of vitamins.

### MIDDLE-CLASS HEALTH INSURANCE.

IN the interesting and valuable paper on "The Need for Extended Sickness Insurance," which he read before the Insurance Institute of London on November 17, Lord Dawson of Penn dealt with a subject that is certain to bulk largely in the public mind in the near future. Since the war the conditions of life for the middle class have changed seriously to their detriment. In every case their expenses have gone up substantially and, even where incomes have increased, their growth has been altogether disproportionate to the additional claims. The dearth of servants and the cost and inefficiency of those that are obtainable have thrown a very trying strain upon wives and daughters—a strain which is perilously near breaking-point when illness or a new baby enters the house. The cost of illness, like the cost of everything else, has increased, and some method of insuring against it has become as important for the professional classes as for those already provided for by the existing Health Insurance arrangements. We are able now to insure against every other imaginable contingency, yet, as Lord Dawson truly says, there is "no direction in which the need is greater and the performance less than in security against illness." The difficulties of such insurance are manifold, and so distinguished an actuary as Sir Alfred Watson has to confess that he cannot yet see his way to devising a plan and fixing a rate of premium which would enable an insurance company to transact such business with safety. Yet we may be sure that, sooner or later, the obstacles will be surmounted.

### HOSPITALS AND COOKS.

THERE is little room for surprise that complaints should be made of hospital cooking when it is officially stated that the kitchen of the Tooting Bec Hospital of the Metropolitan Asylums Board, which will shortly be supplying food for 2,600 people daily, is "entirely without skilled supervision," both the head cook and the assistant cook having recently resigned. Resignation, in fact, is the fashion in these hospitals, and 145 advertisements for cooks have been published during a year which is not yet over. The difficulty is by no means new, and even when qualified cooks have been obtained it has been found impossible to retain them for any length of time. Many hospitals in and out of London have had similar troubles. Only too often exorbitant wages are asked by inefficient people who, in the interests of the patients and staff, have had to be dismissed when their inefficiency became too patently obvious. It begins to look as though the hospitals would have to band themselves together to establish a hospital school of cookery, and even then there would be no certainty that the experts whom it turned out would continue to be satisfied. The work in a hospital kitchen is necessarily hard and continuous, but the pay is usually good. Some part of the difficulty is probably traceable to post-war dislike of hard work, even when combined with good pay. That is a trouble which may be expected in great measure to cure itself when the country has more nearly settled down after the great upheaval, but in the meantime it is deplorable that patients and nurses should suffer.

### GRAPHIC HEALTH TEACHING.

ONE of the great lessons taught, or rather retaught, by the British Empire Exhibition has been the value of models and illustrations for teaching purposes. Adults and children alike have thronged to see the objects about which they have perhaps read for years, and they have taken away a more lasting impression than that produced by even long and laborious book study. The Tropical Health exhibit in the Government building was a notable example of the extraordinarily graphic and fascinating way in which disease and the statistics of disease, though at first these things sound dry and dreadful, can be presented to the layman. It sounds incongruous to speak of statistics as interesting, but they were made so by means of a uniform illuminated device which arrested the attention and informed the mind at a glance. It was interesting to note that in the whole group of twenty-seven diseases dealt with there was not one which could not, to a very large extent, be prevented by wisely conceived and carefully executed hygienic measures. We were reminded by those who were responsible for the exhibit that it is not sufficient for any of us to rest content in our own personal safety, but that we must adopt the broader view and help to stamp out disease for the vast native populations within the Empire and to dry up the reservoirs of infection.

### PASSING THE WART.

IN a recent number of the chief Swiss medical journal, *Schweizerische Medizinische Wochenschrift*, Dr. Bonjour, of Lausanne, has published a paper in which he shows, with the help of numerous photographs and diagrams, that though warts may not be a figment of the imagination, they may certainly be made to come and go by purely psychic means. All the world over it has been the practice from time to time for persons with warts to pass them on to their neighbours simply by applying the warts to the neighbour's skin and saying, "I pass my warts on to you." The result is that the warts atrophy on the donor's skin, while they sprout out on the skin of the recipient of this Danaic gift. In fact, "passing the wart" would seem to be the lineal ancestor of a custom well known during the Great War as "passing the buck." Fortunately, as Dr. Bonjour points out, it is not necessary to inflict one's own warts on someone else in order to get rid of them. He claims that for the past thirty years he has been treating warts by suggestion, and that only in two cases has he failed. His procedure is perfectly simple. The owner of a warty hand places it on a sheet of paper and its outline is traced with a pencil. The warts are marked in their natural size on this drawing, the patient is then blindfolded, and when his hand is stretched out each wart is lightly touched with a finger or rod by someone else who says: "From to-day you will feel your warts no more; they will disappear and you must not touch them more." Simple as this ritual is, it can be further simplified. Dr. Bonjour has never aimed to cure warts, even when he has undertaken the treatment in the course of a social entertainment or by correspondence. The scepticism of the unhappy possessor of warts does not render this treatment futile, and even when they are multiple and of old standing, they ultimately atrophy and disappear under treatment by suggestion. Warts are not of supreme importance to mankind, but if they can be exploited as a means of testing scientifically the influence of mind on matter, they should prove much more interesting to physicians in general and psychologists in particular than they have been hitherto.

### SURPRISE VISITS TO MENTAL HOSPITALS.

The Wiltshire County Mental Hospital has issued a letter to all Boards of Guardians for the better information of any not aware of the circumstances in which these visits take place. Delegations from the Guardians visit the mental hospitals to see and interview the patients from their Union. A notice of such visits is usually sent in advance; the patients are collected together, and, as a rule, the Medical Superintendent or his representative and certain attendants are present, but, this letter insists, "no obstacle is raised to a private interview if desired." The reasons for the collection of the patients together, and for the presence of the mental hospital's staff, are practical. Asylums are often huge institutions; the patients are classified on medical grounds, not according to the Union whence they come; their condition may be such as to render an experienced person a necessary intermediary.

### THE LETTER AND THE SPIRIT.

All this is plain to anyone who has visited a mental hospital and observed the condition of the patients and the size of the institutions. Moreover, the Committee of the Wilts Asylum expressly adds that it "has no objection to surprise visits by deputations duly appointed." There is, on the face of it, nothing more to be said, and the lay Press misleads the public whenever it attempts to make capital out of these reasonable arrangements. Yet, it must always be remembered, the best regulations in the world depend for their virtue on the spirit in which they are carried out. If any guardian, visiting any mental hospital, is in doubt about this spirit, his proper course is to be appointed to make a surprise visit to secure a private interview. But he will defeat the end he has in view if he conducts or acts upon this interview otherwise than he would wish to be treated were the Medical Superintendent visiting officially his own home.

